# MiR-301a-5p/SCIN promotes gastric cancer progression via regulating STAT3 and NF-κB signaling

**DOI:** 10.7150/jca.59747

**Published:** 2021-07-06

**Authors:** Yingying Huang, Xiaoxiao Du, Xiangliu Chen, Chuanzhi Chen, Haiyong Wang, Yan Yang, Lisong Teng

**Affiliations:** 1 Department of oncological surgery, The First Affiliated Hospital, College of Medicine, Zhejiang University.; 2 Cancer Institute (Key Laboratory for Cancer Intervention and Prevention, China National Ministry of Education, Zhejiang Provincial Key Laboratory of Molecular Biology in Medical Sciences), The Second Affiliated Hospital, Zhejiang University School of Medicine, China.

**Keywords:** MicroRNA, miR-301a-5p, gastric cancer (GC), progression, EMT

## Abstract

**Objective:** Gastric cancer (GC) is a type of highly malignant cancer. Although the diagnostic and therapeutic methods are innovating, the outcome of GC patients is still poor. Therefore, our research was carried out to explore potential molecular mechanism in the diagnosis of GC.

**Materials and methods:** Bioinformatics analyses were used to obtain microRNA and target mRNA of interest. The expression level of miR-301a-5p and Scinderin (SCIN) mRNA were detected by quantitative real-time PCR (qRT-PCR). Western blot assay was used to investigate SCIN protein level. Cell Counting Kit-8 assay (CCK-8) and colony formation assay were used to investigate cell proliferation ability. Transwell assay was employed to examine cell motility. The interaction between miR-301a-5p and SCIN mRNA was verified by dual-luciferase reporter assay.

**Results:** The qRT-PCR analysis revealed that the expression of miR-301a-5p was higher in gastric cancer tissues than para-cancer tissues (P<0.05). Cox regression analysis showed upregulated miR-301a-5p was associated with larger tumor size (P=0.036) and more advanced TNM stage (P=0.048). The Kaplan-Meier analysis showed a correlation between increased miR-301a-5p expression and shorter overall survival (OS)(P=0.018). By using bioinformatic analysis, SCIN was predicted as one of the targets of miR-301a-5p. Overexpressing miR-301a-5p promoted proliferation and motility of GC cells while knockdown of SCIN exhibited the same performance. Further, we verified the alteration of miR-301a-5p and SCIN expression level could affect the epithelial-mesenchymal transition (EMT) progression on GC cells via STAT3 and NF-κB signaling.

**Conclusion:** Highly expressed miR-301a-5p was associated with aggressiveness of GC. Upregulation of miR-301a-5p promoted malignant phenotype of GC by targeting SCIN. The present results indicated miR-301a-5p might be a promising molecule in the prognosis of GC.

## Introduction

Gastric cancer (GC) is the fifth most common cancer with high cancer-related mortality around the world^1^. The mortality of gastric cancer among cancer-related death has been the second place in China^2^. Although there are various treatments for gastric cancer, the five-year survival rate of patients with advanced GC is still frustrating^3^. Thus, further studies on GC pathogenesis and identifying predictive biomarkers of GC patients are in at imminent.

MicroRNAs(miRNAs) is a type of non-coding RNA with about 22 nucleotides which negatively regulate gene expression by interacting with the 3'-untranslated regions (3'-UTR) of target mRNAs and suppressing translation or inducing degradation^4^. It has been reported that microRNAs displayed pleiotropic functions in cellular progresses involved in carcinogenesis and a large number of researches showed that aberrantly expressed microRNAs played diversified roles in various tumors^5^, ^6^. MiR-301a-5p is regarded as an important member of miRNAs family in studies^7^, ^8^. Overexpressed miR-301a-5p could rescue the depression in proliferation, migration and invasion of hepatocellular carcinoma (HCC) cell which were induced by down-regulation of lncRNA EPB41L4B-AS2^7^. Hypoxic tumor-derived exosomal miR-301a regulated M2 macrophage polarization and further promoted pancreatic cancer metastasis by PTEN/PI3Kγ pathway^9^. In another study, miR-301a acted as NF-κB activator in pancreatic tumor cell^10^. Upregulated expression of miR-301a in prostate cancer and breast cancer tissues indicated poor prognosis^11^, ^12^. Also, miR-301a played a role in reducing intestinal epithelial integrity and promoting intestinal inflammation, thus leading to colitis-associated cancer development by depressing BTG1 expression^13^.

MicroRNAs modulate cellular progresses by regulating targeted genes. Scinderin (SCIN), a type of Ca^2+^-dependent actin-severing and -capping protein, is required for megakaryocyte differentiation, maturation, polyploidization and apoptosis with the release of platelet-like particles^14^. Another study found that SCIN together with Cdc42 performed important function in the migration and invasion of melanoma cell^15^. Human bladder cancer cell line HT1376 with high SCIN expression showed significant cisplatin resistant and SCIN mRNA knockdown reduced cell proliferation with mitochondria-mediated apoptosis^16^. Therefore, SCIN is important for regulating cancer cell survival and migration.

In the present study, we investigated the relationship between miR-301a-5p and SCIN and their regulatory effects via STAT3 and NF-κB signaling on the proliferation and motility of gastric cancer cell lines. These results can help to explore novel biomarkers and therapeutic approaches to GC.

## Materials and methods

### Patients and samples

Gastric cancer tissue and para-cancer tissue samples were randomly collected from 45 patients undergoing gastric cancer surgery between 2010 and 2015 in the First Affiliated Hospital of Zhejiang University, Hangzhou, Zhejiang Province, China. All patients had not received radiotherapy or chemotherapy before radical gastrectomy and follow-up information was available for all patients. All the samples were pathologically diagnosed as gastric cancer. Patients were informed that specimens were stored by the hospital and used for scientific research potentially. Patients signed informed consent to participants was exempted by the Clinical Research Ethics Committee of the First Affiliated Hospital of Zhejiang University (Approval No:2020-766). The study was conducted in accordance with Declaration of Helsinki and its amendments.

### Bioinformatics Analysis

The miRNAs and mRNAs expression profiles of stomach adenocarcinoma (STAD) were firstly downloaded from GEO Database (http://www.ncbi.nlm.nih.gov/geo/), and the differential analysis was performed, the threshold was set as absolute value of log2 fold change >1, adjusted p value <0.05. The miRNAs expression level of gastric cancer and normal gastric tissues was reconfirmed by TCGA data (http://www.cancer.gov/tcga). Target genes of miRNA were predicted in TargetScan (http://www.targetscan.org/), and the final target gene was determined by intersecting with differential expression mRNAs.

### Cell culture and transfection

GC cell lines AGS, HGC-27, MGC803, SGC7901, BGC823, SNU-1, NCI-N87, MKN-45 were purchased from the Cell Bank of the Chinese Academy of Sciences (Shanghai, China) and human non-malignant gastric mucosal cell line GES-1 was obtained from the China Center for Type Culture Collection (CCTCC). All the cell lines were cultured according to their protocols: AGS, HGC-27, MGC803, SGC7901, BGC823, SNU-1, NCI-N87, MKN45 were cultured in RPMI1640 medium with 10% fetal bovine serum sand 100u/ml penicillin/streptomycin, GES-1in DMEM medium with 10% fetal bovine serum and 100u/ml penicillin/streptomycin in cell incubator with 5% CO2 at 37℃.

Cells were transfected with 50nM miRNA mimics or 75nM inhibitor using lipofectamine 3000 (Invitrogen, USA). Interfering RNAs (siRNA) targeting SCIN was obtained from Genepharma (Shanghai, China) (siRNA1: 5'-GGUGAGAGCCACAGAAGUUTT-3'; siRNA2: 5'-GGAGCAGAGUAUGUAGCAATT-3'; siRNA3: 5'-GGACACCAAUUGUCAUCAUTT-3') and transfected at concentration of 50nM. Random sequence of RNA duplex was used as negative control for miRNA and siRNA. Plasmids expressing sh-SCIN or sh-NC were purchased from Genepharma (Shanghai, China).

### Quantitative real-time polymerase chain reaction (qRT-PCR)

Total RNA was extracted from cells and tissues by using Trizol (Invitrogen) followed by RT-PCR reaction. MiR-301a-5p was reverse-transcribed using Mir-X miRNA First-Strand Synthesis Kit (Clontech, Osaka, Japan) and measured using TB Green® Premix Ex Taq™ II (Takara, Osaka, Japan) via StepOne Real-Time PCR System (Applied Biosystems, Foster City, CA, USA). U6 RNA was used as miRNAs endogenous control and target gene mRNA level was normalized to GAPDH expression. MiR-301a-5p and SCIN specific primers were purchased from Tsingke (miR-301a-5p GCTCTGACTTTATTGCACTACT; SCIN FW 5'-ATGGCTTCGGGAAAGTTTATGT-3', SCIN RW 5'-CATCCACCATATTGTGCTGGG-3'). Relative expression analysis was performed using the comparative CT method (2^-ΔΔCT^).

### Dual-luciferase reporter assay

To verify the target sites of miR-301a-5p and SCIN, HEK-293T cell were co-transfected with miR-301a-5p mimics or control and luciferase reporter constructs containing wild-type or mutated SCIN 3'-UTR. Cells were split and luciferase activity was measured using Dual-Luciferase Reporter Assay Kit (Promega, Madison, WI, USA).

### Western blotting assay

Total proteins were extracted from GC cells and tissues, and their concentrations were measured by Pierce™ Rapid Gold BCA Protein Assay Kit (Thermo Fisher Scientific). Equal amounts of total protein sample from various samples were separated by SDS-PAGE and transferred onto PVDF (polyvinylidene fluoride) membrane (Millipore, USA). The specific primary antibodies used in western blotting assay were as follow: SCIN (Abcam, #191396); E-Cadherin (CST, #3195S); N-cadherin (Abcam, #ab76011); ZEB1 (Abcam, #ab203829); phospho-IκBα (Ser32/36) (CST, #9246), IκBα (Abcam, #32518); SNAI1 (CST, #3879); STAT3 (Abcam, #68153); phospho-STAT3 (S727) (Abcam, #131103); NF-κB p65 (Abcam, #32536); NF-κB p65 (phospho-S536) (Abcam, #76302); GAPDH (CST, #3683).

### Cell growth assay

Transfected cells and control cells were seeded in 96-well plates at a density of 1×10^3^ cells/well and incubated at 37℃. Cell viability was analyzed at 6h, 24h, 48h and 72h using Cell Counting Kit-8 (CCK-8, Dojindo, Kumamoto, Japan) according to the manufacturer's protocol.

### Cell migration and Transwell assay

Transwell (8μm with 24 cluster Plate) (Corning, USA) were coated with 50μL of Matrigel (BD, USA)/PRMI-1640 medium (1:14, v/v) for 30 min at 37℃. A total of 2×10^4^ cells were seeded into the upper chambers with 100μL serum-free RPMI-1640 medium while the lower chambers were filled with 700μL RPMI-1640 medium containing 10%FBS. After 48h incubation, the chamber membranes were fixed with 4% paraformaldehyde for 15 min. Cells on the upper surface of chamber membranes were removed with cotton swabs, and the cells on the lower surface of membranes were stained with crystal violet dye (Beyotime, China). The number of invading cells were counted in 5 random visual field and photographed under microscopic.

### Statistical analysis

Data are presented as the mean ±standard deviation (SD) from at least three independent experiments. Student's t tests were performed to analyze the differences between two groups and the Pearson χ^2^ tests were used for binary analysis by using IBM SPSS Statistics software, version 21. P values <0.05 were considered statistically significant.

## Results

### Expression level of miR-301a-5p is upregulated in gastric cancer tissues and cell lines

**Figure 1:**
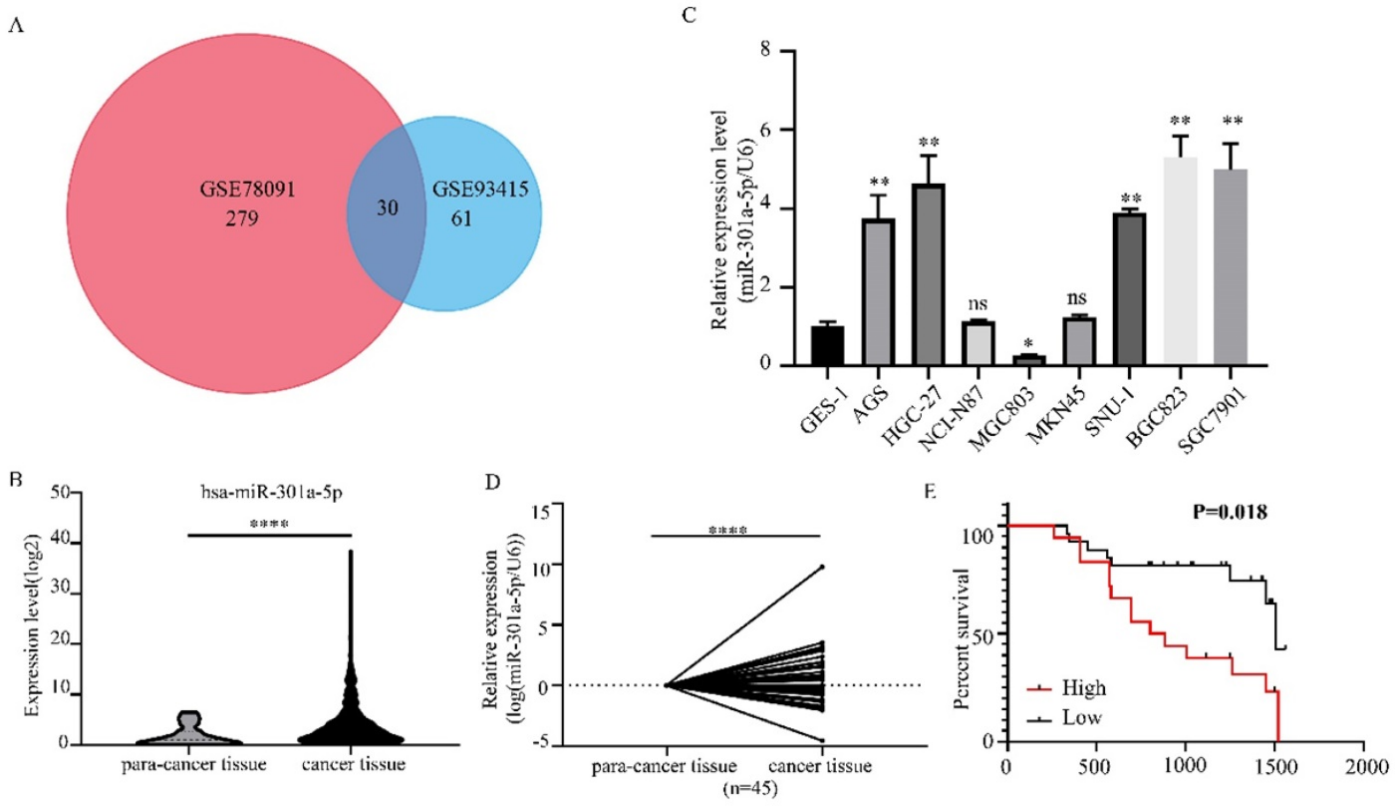
Expression level of miR-301a-5p is upregulated in GC tissues and cell lines. (a) MiR-301a-5p was selected as target miRNA from GEO database and its expression level was altered in GC. (b-d) Expression of miR-301a-5p was increased in GC through verification of TCGA database, GC cell lines and GC samples. U6 was used as an internal control. (e) Patients with high miR-301a-5p expression had shorter overall survival (OS) (P=0.018). * p<0.05, ** p<0.01, *** p<0.001, **** p<0.0001.

We obtained 2 miRNAs expression profiles from GEO database (GSE78091 and GSE93415) and found that miR-301a-5p expression level was altered in GC (|logFC|>1, adjusted P value<0.05) (Figure 1a). Then, we reconfirmed the miR-301a-5p level of gastric cancer tissue and normal gastric tissue from TCGA-STAD data, it was upregulated in GC (Figure 1b). Also, the level of miR-301a-5p in GC cell lines was determined, using GES-1 (a type of human gastric mucosal cell) as comparison (Figure 1c). Furthermore, we obtained 45 pairs GC patient tissues and performed qRT-PCR to measure the expression state of miR-301a-5p (Figure 1d). Table 1 listed the clinical characteristics of 45 patients and their relationships with miR-301a-5p expression. High expression of miR-301a-5p was significantly associated with larger tumor size (P=0.036) and more advanced TNM stage (P=0.048), but had no significant correlation with age and gender (Table 1). The correlation between miR-301a-5p expression and overall survival was analyzed by Kaplan-Meier analysis. The results showed that patients with increased expression of miR-301a-5p had shorter OS than patients with low level of miR-301a-5p (P=0.018 by log rank) (Figure 1e). The above results indicated miR-301-5p was elevated in GC tissues and GC cell lines and was associated with poor prognosis.

### Upregulation of miR-301a-5p promotes proliferation, migration and invasion in vitro

**Figure 2:**
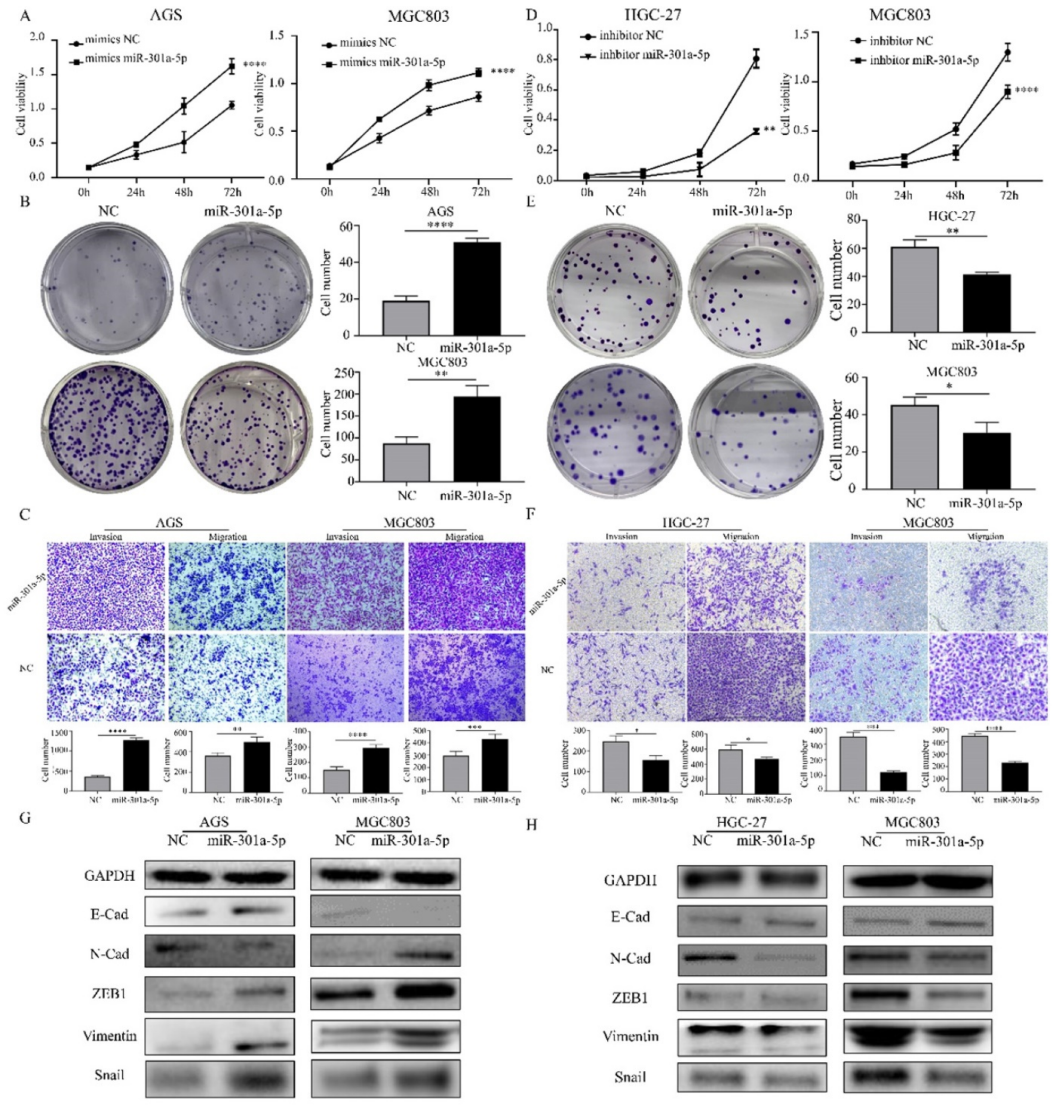
Increasing of miR-301a-5p promotes proliferation, migration and invasion in vitro, vice versa. (a-b) Cell proliferation was determined by CCK-8 and colony formation assay after transfection with mimics of miR-301a-5p. (c) Cell migration and invasion were assessed by Transwell assay after transfection with mimics of miR-301a-5p (magnification, 40×). (d-e) Cell proliferation was determined by CCK-8 and colony formation assays after transfection with inhibitor of miR-301a-5p. (f)Cell migration and invasion were assessed by Transwell assays after transfection with inhibitor of miR-301a-5p (magnification, 40×). (g-h) EMT markers of cell lines were measured after transfection with mimics and inhibitor of miR-301a-5p. * p<0.05, ** p<0.01, *** p<0.001, **** p<0.0001.

The functional roles of miR-301a-5p in the proliferation, migration and invasion of GC cells, which had important impact on tumorigenesis and metastasis, were investigated. We examined the effects of miR-301a-5p overexpression on proliferation and metastasis on AGS and MGC803 cell lines. The results of CCK-8 and colony formation assay showed that when transfecting cells with mimics of miR-301a-5p, cell viability was significantly increased and cell growth was promoted (Figure 2a, b). Meanwhile, the migration and invasion ability were increased (Figure 2c). We also transfected cells with inhibitors of miR-301a-5p to restrain the function of miR-301a-5p. As results, cell viability, proliferation, migration and invasion ability were reduced (Figure 2d-f). Additionally, expression of EMT-related proteins altered when miR-301a-5p level changed (Figure 2g, h). These results indicated that miR-301a-5p as an oncogene could promote proliferation, migration and invasion of GC cells.

### MiR-301a-5p targets SCIN and SCIN is downregulated in GC tissues and cell lines

**Figure 3:**
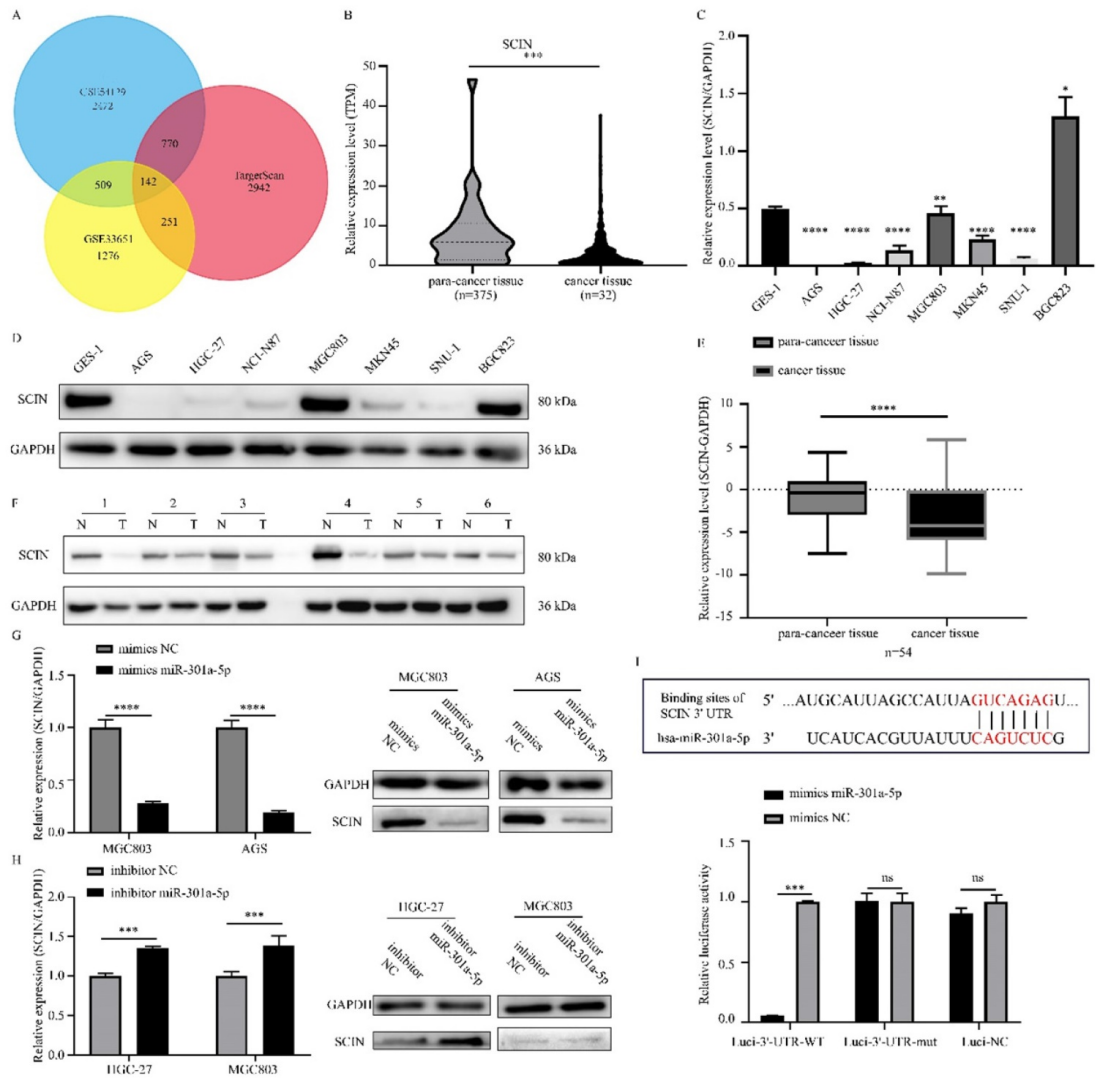
Direct regulation of SCIN by miR-301a-5p in gastric cancer. (a) SCIN was predicted as target as miR-301a-5p via bioinformatic analyses. (b) Expression level of SCIN in TCGA database. (c-d) mRNA and protein expression level of SCIN in GC cell lines by qPCR and western blot. GAPDH was used as internal control. (e-f) SCIN mRNA and protein level of GC patient sample was determined by qRCR and western blot. (g) After transfecting mimics of miR-301a-5p in cell lines, the alteration of SCIN mRNA and protein were measured by qPCR and western blot. GAPDH was used as internal control. (h) After transfecting inhibitor of miR-301a-5p in cell lines, the alteration of SCIN mRNA and protein were measured by qPCR and western blot. GAPDH was used as internal control. (i) miR-301a-5p binding sites in the 3'-UTR of SCIN mRNA. Dual luciferase reports assays exhibited that miR-301a-5p markedly suppressed luciferase activity of HEK-293T carrying wild-type reporter constructs. * p<0.05, ** p<0.01, *** p<0.001, **** p<0.0001.

Since miRNAs modulating cellular progresses by targeting downstream genes, we explored the target genes of miR-301a-5p. Online miRNAs analysis software (TargetScan) was used to predict the downstream mRNA of miR-301a-5p. Otherwise, we downloaded 2 mRNA expression profiles (GSE33651 and GSE54129) from GEO database and gained a gene set of downregulated genes with threshold of |logFC|>1 and adjusted P value<0.05. The expression sets and the predicted target gene set were intersected to obtain SCIN as interested targeted gene (Figure 3a). SCIN is a type of Ca^2+^-dependent actin-severing and -capping protein and mainly located in cytoplasm and cytoskeleton structure^17^. Then, we verified the SCIN expression level based on TCGA-STAD database (Figure 3b), qRT-PCR and western blot assay results of cell lines showed that SCIN expressed at low level in AGS and HGC-27, but comparative high level in MGC803 (Figure 3c-d). 45 pairs of GC cancer tissue and para-cancer tissue samples were examined, the SCIN mRNA level was significantly down-regulated in tissues of GC patients (Figure 3e). Meanwhile, the expression level of SCIN protein extracted from GC patients was tested (typical pairs of tissues were showed) (Figure 3f). Further, we transfected MGC803 and AGS with mimics of miR-301a-5p and measured SCIN expression status by qRT-PCR and western blot, the miRNA level was sharply elevated while SCIN mRNA and protein expression were reduced (Figure 3g). Inversely, miRNA level was reduced and SCIN increased at the present of inhibitor of miR-301a-5p (Figure 3h). To further prove that miR-301a-5p works by targeting the 3'-UTR of SCIN mRNA, dual-luciferase reporter assay was performed. And results demonstrated that there was an acting site between miR-301a-5p and 3'-UTR of SCIN mRNA (Figure 3i). The above results showed SCIN was the target of miR-301a-5p and its expression level was affected by miR-301a-5p.

### SCIN acts as tumor suppressor in GC

**Figure 4:**
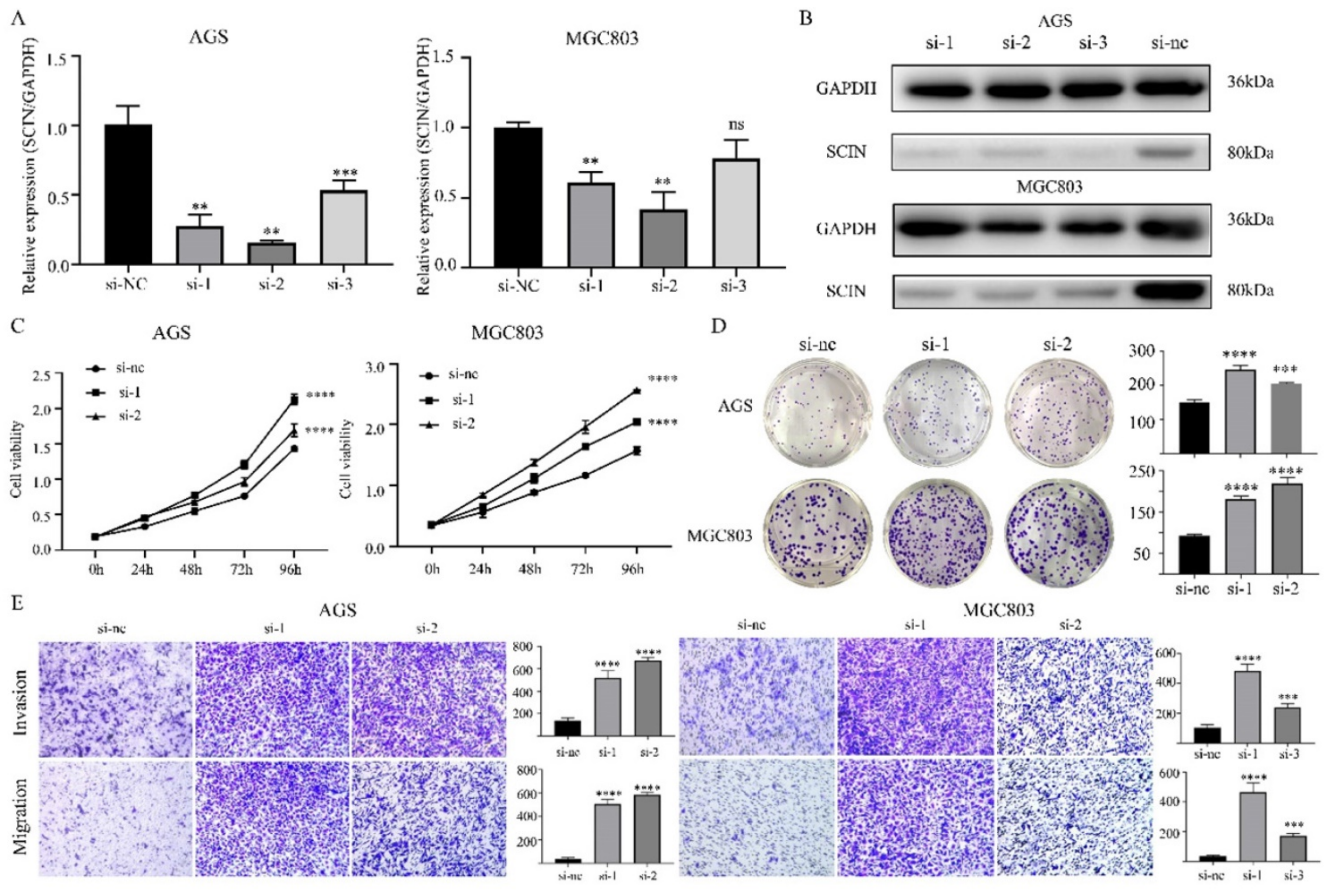
Down-regulation of SCIN promotes GC cells proliferation, migration and invasion in vitro. (a-b) Knockdown efficiency of SCIN in AGS and MGC803 was confirmed by qPCR and western blot. (c-d) Cell proliferation was determined by CCK-8 and colony formation assays after transfected with SCIN siRNA. (e) Cell migration and invasion were assessed by Transwell assays after transfection with SCIN siRNA (magnification, 40×). * p<0.05, ** p<0.01, *** p<0.001, **** p<0.0001.

**Figure 5:**
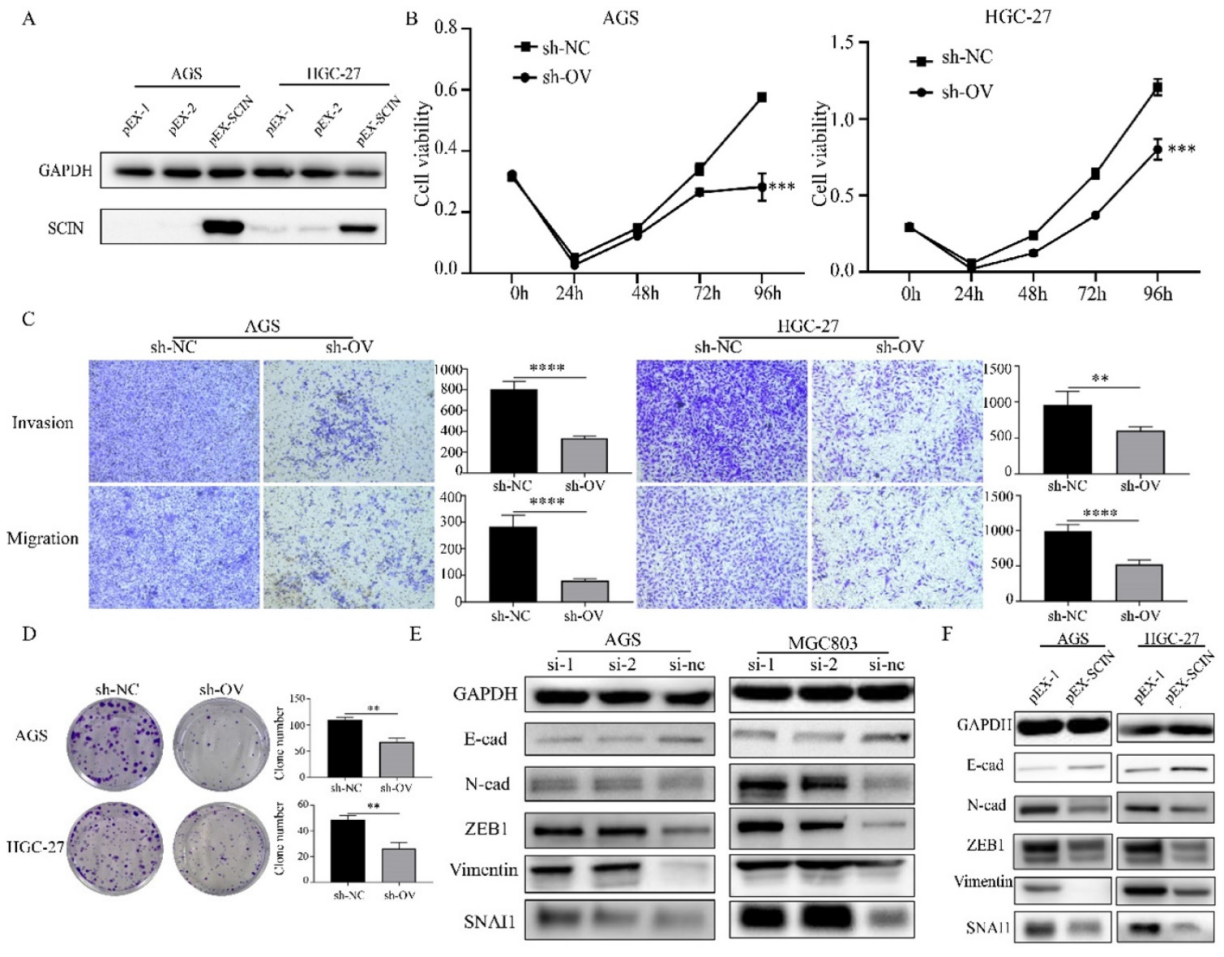
Overexpression of SCIN reduces GC cell proliferation, migration and invasion ability in vitro. (a) Overexpression efficiency of SCIN in HGC-27 and MGC803 was confirmed by western blot. (b, d) Cell proliferation was determined by CCK-8 and colony formation assays after transfected with SCIN sh-RNA. (c) Cell migration and invasion were assessed by Transwell assays after transfection with SCIN sh-RNA (magnification, 40×). (e-f) EMT markers in GC cell were measured after transfection with SCIN siRNA or sh-RNA. * p<0.05, ** p<0.01, *** p<0.001, **** p<0.0001.

In order to further investigate the function of SCIN, we induced SCIN overexpression and knockdown in GC cell lines. Firstly, we transfected GC cell lines with siRNA to depress the expression of SCIN, qRT-PCR and western blot were used to verify the knockdown efficiency of siRNAs (Figure 4a-b). SCIN-depleted cells exhibited significant decreased proliferation, colony formation, migration and invasion abilities (Figure 4c-e). Furthermore, we transfected GC cells with overexpression plasmid and the efficiency of transfection was confirmed by western blot assay (Figure 5a). Conversely, overexpression of SCIN led to opposite results on cell proliferation, colony formation and ability of migration and invasion (Figure 5b-d). We also observed the level of EMT-related markers has changed with the alteration of SCIN. SCIN-knockdown group showed an increasing of mesenchymal markers while SCIN-overexpression group had decreasing trend of mesenchymal markers (Figure 5e-f). These results indicated SCIN functioned as a tumor suppressor by regulating EMT progression in GC cells.

### MiR-301a-5p targeted SCIN and promoted EMT progression by activating STAT3 and NF-κB signaling

**Figure 6:**
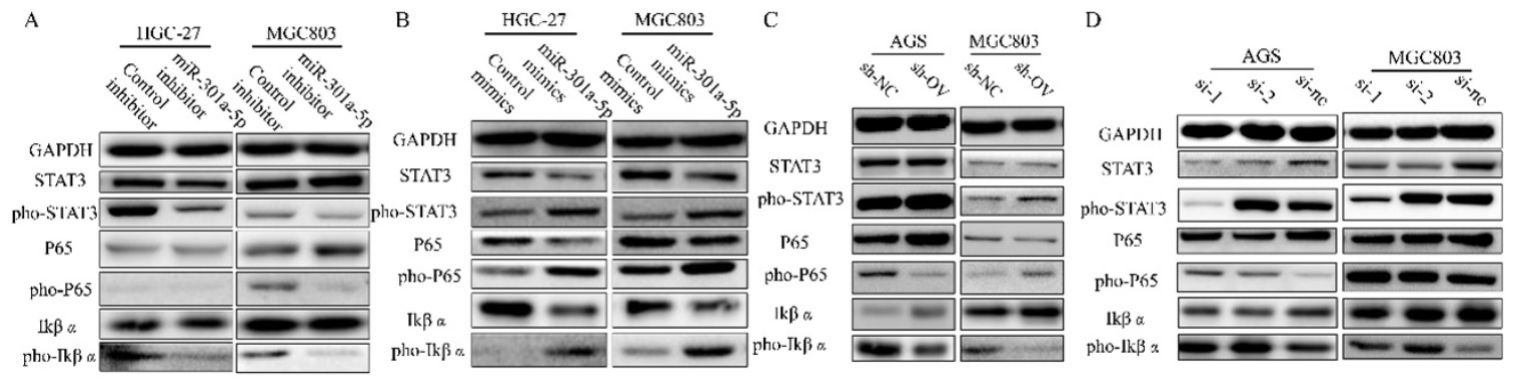
STAT3 and NF-κB signaling are activated by overexpressing miR-301a-5p or down-regulating SCIN. (a-b) STAT3 and NF-κB signaling-related proteins which were extracted from HGC-27 and MGC803 after transfection with mimic or inhibitor of miR301a-5p were measured by western blot assay. (c-d) STAT3 and NF-κB signaling-related proteins which were extracted from AGS and MGC803 after overexpression or knockdown of SCIN were measured by western blot.

In the previous results, we have confirmed that high level of miR-301a-5p was related to more proliferative ability and higher motility of GC cells. Meanwhile, western blot results showed expression of EMT-related proteins altered when transfecting cells with miR-301a-5p mimics or inhibitors (data was showed before). Whereafter, we analyzed protein expression of key signaling and found NF-κB signaling was activated by inducing higher level of miR-301a-5p in GC cells. Since researchers have confirmed that continuously activated STAT3 could maintain the continuous activation of the NF-κB signaling pathway in tumors^18^, we tested STAT3 and NF-κB as well as their active forms of phosphorylation (Figure 6a-b). When changing the expression level of SCIN, the adjustment of activation of STAT3 and NF-κB signaling displayed the same tendency (Figure 6c-d). Our data suggest that miR-301a-5p acts as an oncogene by suppressing the expression of SCIN and regulating gastric cancer progression via STAT3 and NF-κB signaling.

## Discussion

Accruing data has shown that miR-301a-5p played momentous roles in tumor progression as tumor suppressor or oncogene 7, 8, 19, 20. MiR-301a was verified upregulated in GC and associated with progression and poor prognosis^21^. In the present study, we found that miR-301a-5p significantly increased in both GC tissues and cell lines. Also, it promoted GC cell proliferation, migration and invasion ability in vitro. Furthermore, SCIN was confirmed as a functional target of miR-301a-5p.

MicroRNAs is a category of short, non-coding RNA with about 22 nucleotides which could act as mRNA sponge and suppress target gene translation or inducing its degradation^4^. What's more, microRNAs are involved in various pathogenesis including cancer and play central roles in competing endogenous RNAs (ceRNAs) network^22^. Thus, it's acceptable for us to hypothesize that miRNAs might be a potential diagnostic marker and promising therapeutic target in cancers. In the present study, we validated that miR-301a-5p was upregulated in gastric cancer tissues and cell lines and the clinical data showed that increasing of miR-301a-5p was significantly correlated with advanced TNM stage and tumor size, suggesting that miR-301a-5p might be involved in GC progression. Also, Kaplan-Meier analysis showed that high expression level of miR-301a-5p was correlated with shorter overall survival of patients. Moreover, we found interfering with the expression of miR-301a-5p dramatically inhibited proliferation, migration and invasion ability in HGC-27 and MGC803 cells, while overexpression of miR-301a-5p promoted proliferation, migration and invasion in MGC803 and AGS. These data implicated miR-301a-5p acted as oncogene in gastric cancer.

Non-coding RNAs can be divided into several types based on length. MiRNAs are single-stranded RNAs and play as the negative regulators of their targeted mRNAs by based pairing to 3'-UTR and then targeted genes undergo translation block or mRNAs decay^23^-^25^. In the present study, we used bioinformatics analysis and screened out that SCIN had a complementary sequence to miR-301a-5p in its 3'-UTR. Then, two methods were used to confirm SCIN was the target of miR-301a-5p. Firstly, dual-luciferase reporter assay was performed on HEK-293T cell. And the results showed that miR-301a-5p significantly repressed the luciferase activity in cells transfected with vector carrying wild-type SCIN 3'-UTR but not inhibited the luciferase activity transfected with vector carrying mutation sites. Secondly, ectopic expression of miR-301a-5p in GC cells was performed and the expression of miR-301a-5p was verified by qRT-PCR, meanwhile, we tested the SCIN mRNA and protein expression level and found it was decreased. These results both indicated that high miR-301a-5p and low SCIN expression were found in GC and they may together play significant roles in GC progression.

Although the role of SCIN in cancer has been reported before, the correlation between miR-301a-5p and SCIN are unclear. In the current study, we revealed SCIN was repressed by miR-301a-5p and its repression promoted cell growth and motility in GC cells via activating the STAT3 and NF-κB signaling. Furthermore, the mechanistic interaction between miR-301a-5p and SCIN in regulation of gastric cancer cell phenotype was revealed.

STAT3 and NF-κB are two considerable factors controlling the ability of tumorigenesis and malignant transformation including anti-apoptosis, tumor angiogenesis and invasiveness^26^, ^27^. Activation and interaction of STAT3 and NF-κB signaling in cancers has been investigated extensively and STAT3 activation could prolong NK-κB continuous activation^18^. Our results showed that ectopic expression of miR-301a-5p or interference expression of SCIN in GC cells gave rise to activation of STAT3 and NF-κB signaling, and EMT markers showed mesenchymal markers expression were increased at the same time. And inhibiting miR-301a-5p or ectopic expression of SCIN presented decreasing expression of mesenchymal markers.

## Conclusion

Our study found that miR-301a-5p was upregulated in GC and significantly associated with advanced tumor stage. Furthermore, we found that miR-301a-5p functioned as sponge and targeted to 3'-UTR of SCIN mRNA, and promoted the proliferation and motility of GC cell. EMT markers changed with miR-301a-5p, and increasing expression of miR-301a-5p made the EMT process tend to develop in the direction of mesenchymal transition. Additionally, miR-301a-5p mediated GC cell phenotype through regulating the activity of STAT3 and NF-κB pathway. Our findings may provide a potential diagnostic and promising candidates for GC therapeutic strategies.

## Figures and Tables

**Table 1:** Correlation between hsa-mir-301a-5p expression and clinical features of the GC patients

Clinical parameter	hsa-mir-301a-5p expression		P value
	high	low	
**Age (years)**			0.436
≥60	11	12	
<60	8	14	
**Gender**			0.478
Male	12	19	
Female	7	7	
**Tumor size**			**0.036**
≥5cm	11	7	
<5cm	8	19	
**TNM stage**			**0.048**
I-II	4	13	
III-IV	15	13	

**Notes:** *Statistical analyses by Pearson's χ2 test or Fisher's exact test **Abbreviation:** gastric cancer: GC
